# Green and Near-Infrared Dual-Mode Afterglow of Carbon Dots and Their Applications for Confidential Information Readout

**DOI:** 10.1007/s40820-021-00718-z

**Published:** 2021-09-16

**Authors:** Yuci Wang, Kai Jiang, Jiaren Du, Licheng Zheng, Yike Li, Zhongjun Li, Hengwei Lin

**Affiliations:** 1 College of Chemistry, Zhengzhou University, Zhengzhou, 450001 People’s Republic of China; 2 International Joint Research Center for Photo-Responsive Molecules and Materials, School of Chemical and Material Engineering, Jiangnan University, Wuxi, 214122 People’s Republic of China; 3 Key Laboratory of Graphene Technologies and Applications of Zhejiang Province, Ningbo Institute of Materials Technology & Engineering (NIMTE), Chinese Academy of Sciences, Ningbo, 315201 People’s Republic of China

**Keywords:** Carbon dots, Dual-mode afterglow, Room temperature phosphorescence, Thermal activated delayed fluorescence, Information security

## Abstract

Near-infrared (NIR), particularly NIR-containing dual-/multi-mode afterglow, is very attractive in many fields of application, but it is still a great challenge to achieve such property of materials. Herein, we report a facile method to prepare green and NIR dual-mode afterglow of carbon dots (CDs) through in situ embedding o-CDs (being prepared from o-phenylenediamine) into cyanuric acid (CA) matrix (named o-CDs@CA). Further studies reveal that the green and NIR afterglows of o-CDs@CA originate from thermal activated delayed fluorescence (TADF) and room temperature phosphorescence (RTP) of o-CDs, respectively. In addition, the formation of covalent bonds between o-CDs and CA, and the presence of multiple fixation and rigid effects to the triplet states of o-CDs are confirmed to be critical for activating the observed dual-mode afterglow. Due to the shorter lifetime and insensitiveness to human vision of the NIR RTP of o-CDs@CA, it is completely covered by the green TADF during directly observing. The NIR RTP signal, however, can be readily captured if an optical filter (cut-off wavelength of 600 nm) being used. By utilizing these unique features, the applications of o-CDs@CA in anti-counterfeiting and information encryption have been demonstrated with great confidentiality. Finally, the as-developed method was confirmed to be applicable to many other kinds of CDs for achieving or enhancing their afterglow performances.

**Supplementary Information:**

The online version contains supplementary material available at 10.1007/s40820-021-00718-z.

## Highlights

- A facile method was developed to achieve visible light (green) and near infrared dual-mode afterglow emissions from carbon dots (CDs)-based materials at ambient conditions for the first time.
- We proposed a promising method in advanced information security applications through a special manner of readout.
- The as-developed method was confirmed to be applicable to many kinds of CDs for achieving or enhancing their afterglow performances.

**Supplementary Information:**

The online version contains supplementary material available at 10.1007/s40820-021-00718-z.

## Graphical abstract

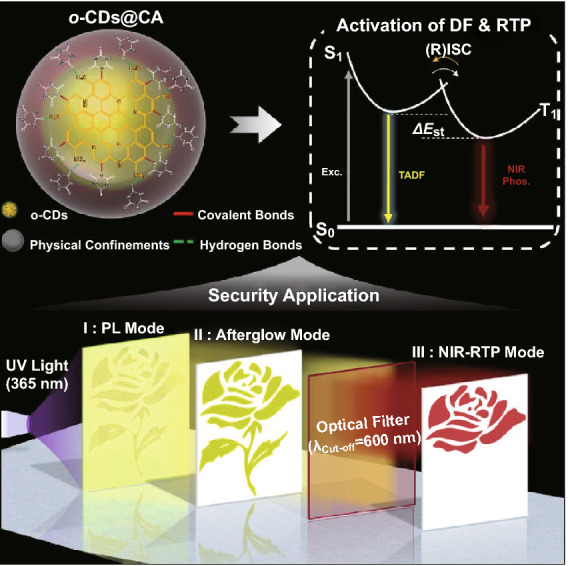

## Introduction

Information encryption and anti-counterfeiting are highly significant in military, civilian and economic fields [1]. In the past decades, printing encrypted information and anti-fake labels using stimuli-responsive luminescent materials have been employed as one of the most popular security technics [2]. In this method, luminescence signal would be changed under a specific external stimulus, preventing the encrypted information or security labels to be stolen, mimicked and/or forged [3, 4]. However, similar luminescence changes are becoming easier and easier to be imitated with the development of materials science, which do not meet the requirements for protecting high-level confidential information any longer [1–4]. Therefore, it is desirable to exploit novel encryption and anti-counterfeiting techniques with higher security [5–7].

Afterglow including room temperature phosphorescence (RTP), thermal activated delayed fluorescence (TADF), and long persistent luminescence (LPL) materials have been taken to apply in information security fields recently due to their specific features [8, 9]. Although the encryption performance could be somewhat improved, the single-mode visible-light-based afterglow is still lack of enough confidentiality. Considering the invisibility/insensitivity of near-infrared (NIR) light to human vision [10, 11], we conceive that it could be an ideal encryption strategy to conceal the NIR afterglow coded information by visible light afterglows. Unfortunately, materials with such properties are very difficult to be prepared and have rarely been reported to date [12].

As a new type of luminescent nanocarbon materials, carbon dots (CDs) have attracted much attention and been widely applied in biotechnology, anti-counterfeiting, optoelectronic device, and photocatalysis areas in recent years due to possessing numerous superior features such as excellent photostability, chemical stability, biocompatibility, and cost-effective preparation [13–20]. More impressively, afterglow phenomena of CDs had also been discovered either through embedding them in certain matrices (e.g., poly(vinyl alcohol) (PVA), inorganic salts, urea/biuret, zeolites, and silica) [21, 22], or preparing special-structured CDs by proper carbon precursors and reaction conditions [23, 24]. In both of the above cases, hydrogen bonding interactions between the emissive units of CDs and matrices/CDs’ frameworks usually play a critical role to stabilize the excited triplet states and so as to activate their afterglows [21, 22, 25, 26]. Although afterglow emission wavelengths from CDs-based materials have been successfully regulated from green to red region [27–31], NIR afterglow from CDs has not yet been reported so far, not to mention the NIR-containing dual-/multi-mode afterglows.

Recalled from our previous study that covalent bonds could be employed as an option to fix and rigidify triplet state species [32]. In comparison with hydrogen bonds, the stronger covalent bonding fixation is beneficial for extending occurrence of afterglow from solid state to dispersion state, and meanwhile for achieving RTP and TADF dual-mode afterglows by appropriately decreasing the energy gap between the singlet state (S_1_) and triplet state (T_1_) (*ΔE*_*ST*_) of CDs [32]. Besides, multiple fixation of triplet states by covalent bonds, hydrogen bonds and physical confinements (e.g., rigid network and nanoscale spaces of matrices) had been confirmed enabling more effectively activating afterglow emissions of CDs [33–35]. Inspired by these results, we suppose that NIR-containing dual-mode afterglow emission from CDs could be obtained by taking proper CDs (with potential NIR afterglow property) and a high-efficient manner to fix them.

To validate our hypothesis, CDs that being prepared from o-phenylenediamine (i.e., o-CDs) were selected as emitters because they could emit NIR phosphorescence (Phos) (although very weak) at low temperature (77 K). Significantly, we developed a very efficient strategy to fix CDs via in situ embedding them into cyanuric acid (CA) matrix. By using such an approach, composite of o-CDs and CA (named o-CDs@CA) can be easily prepared. Interestingly, the o-CDs@CA powder exhibits intense NIR RTP (wavelength of maximum at ~ 690 nm) and relatively weak green TADF (wavelength of maximum at ~ 550 nm) dual-mode room temperature afterglows under the excitation of UV light (e.g., 365 nm). To the best of our knowledge, this is the first example to achieve NIR (or NIR-containing) afterglow from CDs-based materials at ambient conditions. Further studies revealed that the formation of covalent bonds between o-CDs and CA appropriately decreased the energy gap *ΔE*_*ST*_ of o-CDs, of which facilitating both intersystem crossing (ISC) and reversed intersystem crossing (RISC) processes and thus being beneficial for simultaneously producing RTP and TADF. Meanwhile, the triplet states of o-CDs in CA matrix are fixed and rigidified by multiple roles including covalent bonds, hydrogen bonds and physical confinements, so as afterglow emission could be activated by effectively restraining the non-radiation decays of their triplet species. Due to the shorter lifetime and insensitiveness to human vision of the NIR RTP of o-CDs@CA, it is completely covered by the green TADF during directly observing. However, the NIR RTP signal can be readily captured if an optical filter (cut-off wavelength (λ_Cut-off_) of 600 nm) being used. By utilizing these unique features, o-CDs@CA could be applied in advanced anti-counterfeiting and information encryption fields with excellent confidentiality. Finally, the as-developed method was confirmed to be applicable to many other kinds of CDs for achieving or enhancing their afterglow performances.

## Experimental Section

### Materials

Reagent grade of o-phenylenediamine (oPD), L-aspartic acid (AA) and folic acid (FA) were bought from Aladdin Chemicals Co. Ltd (Shanghai, China). Citric acid, ethanolamine, ethanediamine (EDA), polyvinyl alcohol (PVA), methylene chloride, ethanol, phosphoric acid and urea were purchased from Sinopharm Chemical Reagent Co. Ltd (Shanghai, China). Pure cyanuric acid (pCA) was purchased from J&K Chemical Reagent Co. Ltd (Beijing, China). All chemicals were used as received without further purification unless otherwise specified. Deionized (DI) water was used throughout this study.

### Synthesis of o-CDs

o-CDs were prepared according to our previous work [36]. In brief, oPD (1.5 g) was dissolved in 150 mL of ethanol, and then this solution was transferred into Teflon-lined autoclaves. After heating at 180 ºC in oven for 12 h and cooling down to room temperature naturally, bright yellow suspension was obtained. The crude product followed purification with a silica column chromatography using mixtures of methylene chloride and methanol as eluents. After removing solvents and further drying under vacuum, the purified o-CDs could be obtained as yellow powder.

### Synthesis of Experimental Cyanuric Acid (eCA)

Typically, 10 g urea was dissolved in 20 mL of DI water, and the formed transparent solution was transferred into a beaker and heated in a domestic oven for 8–10 min (750 W). The produced crude product was crushed and purified by dispersing it in boiling water (100 mL) and centrifuged at 5000 rpm for 5 min to remove the insoluble components. The pure product was gradually separated out when the solution was cooled down to room temperature and finally dried in a vacuum oven (60 ºC for 12 h).

### Preparation of o-CDs@CA

To prepare the composite materials with different loading amounts of o-CDs (0.5, 1.25, 5, 10, 25, and 50 mg) were added into 20 mL of urea solution (0.5 g mL^−1^) to form a transparent solution. Subsequently, the mixed solution was transferred into a beaker and heated in a domestic oven for 8–10 min (750 W) until the water completely evaporated. The formed block composites were purified by grinding into powder, dispersed by boiled water (100 mL) and centrifuged at 5000 rpm for 5 min to remove the insoluble impurities. Finally, the o-CDs@CA were gradually separated out when the solution was cooled to room temperature and then dried in a vacuum oven (60 ºC for 12 h).

### Preparation of o-CDs#CA

o-CDs#CA were prepared by co-crystallization method [37]. In brief, 400 uL of o-CDs (5 mg mL^−1^ in ethanol) were added into a clear and transparent solution (5 mL of boiled water) of pCA (0.5 g). The mixed solution allowed to cool down to room temperature, and the crystals were gradually separated out within 6 h. The obtained o-CDs#CA crystals were washed with DI water (20 mL) and then subjected to freeze drying.

### Preparation of URTP-CDs

URTP-CDs were prepared according to the previous work [24]. In brief, 4.0 mL of ethanolamine was firstly dissolved in 16 mL DI water, and then 8.0 mL of phosphoric acid was added into the ethanolamine aqueous solution drop by drop with stirring. The formed transparent mixture solution was transferred into a beaker and heated in a domestic oven for 5 min (750 W). Upon cooling, the mixture solidified into a dark brown gel-like solid that can be dissolved by the addition of 40 mL DI water. After being neutralized by sodium carbonate, aqueous solution of the crude product was centrifuged (10,000 rpm min^−1^ for 20 min) and filtered through 0.22 μm membrane filter to remove large or agglomerated particles. The supernatant was collected and subjected to dialysis (MWCO: 1000 Da) for a week. Finally, the URTP CDs were obtained by freeze drying.

### Preparation of AA-CDs

AA-CDs were prepared according to the previous work [29]. Typically, 2.0 mL of ammonia solution was added in 8 mL DI water, and then 1500 mg of L-aspartic acid (AA) was slowly added into this solution with stirring, and the precursor was completely dissolved by ultrasonic treatment for 15 min. The as-formed homogeneous and transparent solution was transferred into a beaker and heated in a domestic microwave oven about 2 min (750 W). After cooling to room temperature, the crude yellow gel-like solid was obtained, and completely dissolved by the addition of sodium carbonate solution. For purifying the CDs, the above aqueous solution was firstly centrifuged (10,000 rpm min^−1^ for 20 min) and filtered through 0.22 μm membrane filter to remove large or agglomerated particles, and then the supernatant was collected and subjected to dialysis (MWCO: 1000 Da) for 3 days. Finally, the purified AA-CDs powder can be obtained by freeze drying.

### Preparation of FA-CDs

FA-CDs were prepared according to the previous work [38]. Briefly, folic acid (FA) (1.0 g) was dissolved DI water (100 mL). After stirring for mixing, the solution was transferred to a poly(tetrafluoroethylene) (Teflon)-lined autoclave (75 mL) and heated at 260 °C for 2 h. After the reaction, the reactor was cooled to room temperature naturally. The obtained dark brown solution was centrifuged under 10,000 rpm min^−1^ for 20 min to remove large or agglomerated particles. Finally, FA-CDs were obtained via freeze drying.

### Preparation of C-CDs

C-CDs were prepared according to the previous work [39]. In brief, 2.0 g of citric acid was dissolved in 10 mL DI water, and then the solution was heated in a conventional microwave oven for 7 min (750 W). After cooled to room temperature naturally, the cluster-like product was re-dissolved in 10 mL DI water and centrifuged (10,000 rpm min^−1^) for 10 min, and the supernatant was dialyzed (1000 Da) against water for 24 h. Finally, C-CDs were obtained through freeze drying.

#### Preparation of CE-CDs

CE-CDs were prepared according to the previous work [40]. In brief, 1.0 g of citric acid and 1.6 mL of EDA were dissolved in 30 mL DI water, and then the solution was transferred into a poly(tetrafluoroethylene) lined autoclave under the heat treatment of 200 ºC for 5 h. The obtained orange solution was centrifuged (10,000 rpm min^−1^) for 10 min, and then the supernatant was dialyzed (1000 Da) against water for 24 h. Finally, CE-CDs were obtained through freeze drying.

#### Calculation of the ***ΔE***_***ST***_

The *ΔE*_*ST*_ of materials are calculated according to Eq. 1 based on their fluorescence (FL) and phosphorescence (Phos) emission spectra:

1 $$\Delta E_{{{\text{ST}}}} = \frac{1240}{{\lambda_{{{\text{FL}}}} }} - \frac{1240}{{\lambda_{{{\text{Phos}}}} }}$$

where *ΔE*_ST_ is the energy gap between the singlet and triplet exited states, *λ*_Phos_ and *λ*_FL_ are the wavelengths maxima of Phos and FL emissions, respectively.

#### Equipment and Characterization

Transmission electron microscopy (TEM) observations were performed on a Tecnai F20 microscope. X-ray photoelectron spectroscopy (XPS) spectra were carried out with ESCALAB 250Xi (Thermo Scientific). Scanning electron microscopy (SEM) was performed on a JEOL FESEM 6700F microscope with a primary electron energy of 3 kV. X-ray powder diffraction (XRD) patterns were recorded on a Rigaku D/max-2000 X-ray powder diffractometer (Japan) using Cu Kα (1.5406 Å) radiation. Fourier transform infrared (FT-IR) spectra were obtained on a Nicolet 6700 FT-IR spectrometer. Photoluminescence (PL), afterglow emission and excitation spectra were measured on a Hitachi F-4600 spectrophotometer at ambient conditions. For the temperature-dependent experiment, the sample was placed in a high temperature fluorescence attachment (Orient KOJI, TAP-02) with temperatures controlled between 298.15 and 523.15 K. UV–Vis absorption spectra were recorded on a PERSEE T10CS UV–Vis spectrophotometer. PL and afterglow lifetimes were measured using Fluorolog 3–11 (HORIBA Jobin Yvon). PL quantum yields (QYs) were measured on a QE-2100 quantum efficiency measurement system (Japan Otsuka Electronics). Photographs of PL and afterglow were taken using a Canon camera (EOS 550) under excitation by a hand-hold UV or LED lamps.

## Results and Discussion

### Preparation of o-CDs@CA

**Fig. 1:**
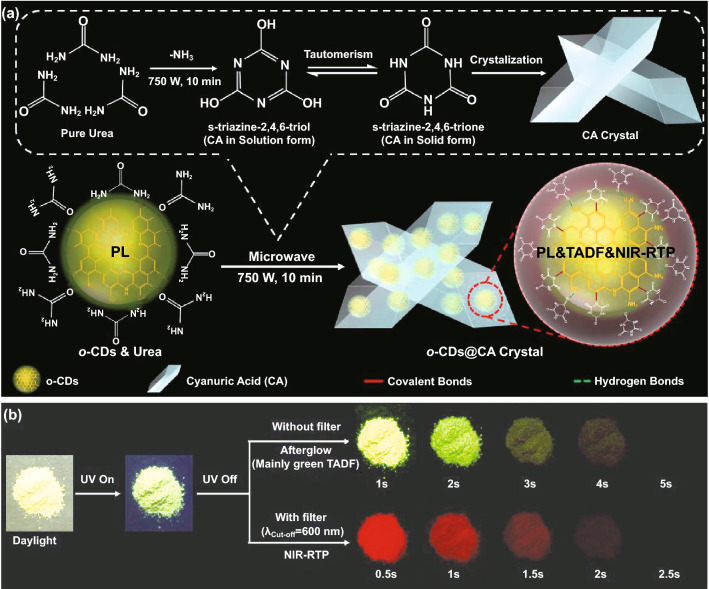
**a** Schematic illustration for the synthesis of CA from urea, in situ embedding and fixing o-CDs into CA matrix, and possible structures of o-CDs@CA; **b** images of the o-CDs@CA powder under daylight, 365 nm UV irradiation, afterglows without and with an optical filter (cut-off wavelength (λ_Cut-off_) of 600 nm) being used

Since the aqueous dispersion of o-CDs emits weak NIR Phos (wavelength of maximum at ca. 690 nm) at low temperature (77 K) under 365 nm ultraviolet (UV) light irradiation (Fig. S1), they are selected as emitters to fabricate NIR-containing dual-/multi-mode afterglow materials. Note that no afterglow emission could be detected at room temperature through embedding o-CDs into matrices that often being used to obtain RTP of CDs (e.g., polyvinyl alcohol (PVA), Fig. S2), indicating that only hydrogen bonding fixation is not sufficient to activate RTP of o-CDs. Herein, we developed a very effective strategy to fix CDs into cyanuric acid (CA) matrix via microwave-assisted heating of the mixture of CDs and urea, in which urea acting as the precursor to in situ produce the host matrix (i.e., CA). Using such an approach, composite of o-CDs and CA (i.e., o-CDs@CA) was easily prepared (Fig. 1a, also see the detailed synthesis procedure in Method section). In order to obtain an optimal photoluminescence (PL) performance, different ratios of o-CDs and urea for preparing o-CDs@CA were screened. As shown in Table S1, sample from the ratio of 0.1% (i.e., o-CDs to urea by weight) exhibits the best PL performance, which is thus used as the optimal formula to prepare o-CDs@CA and the corresponding product is taken to discuss in this study. As expected, the o-CDs@CA powder displays intense green PL emission upon 365 nm UV light irradiation (Fig. 1b), which is well consistent with that of the dispersion of free o-CDs (inset of Fig. S3). Significantly, a bright green afterglow was observed from o-CDs@CA powder after ceasing the UV excitation. The afterglow lasted for a few seconds and could be easily recognized by the naked eye under ambient conditions (Fig. 1b, without filter), even in the form of aqueous dispersion (Fig. S4). More interestingly, a NIR afterglow could be observed if an optical filter (cut-off wavelength (λ_Cut-off_) = 600 nm) being used (Fig. 1b, with filter).

### Structure of o-CDs@CA

**Fig. 2:**
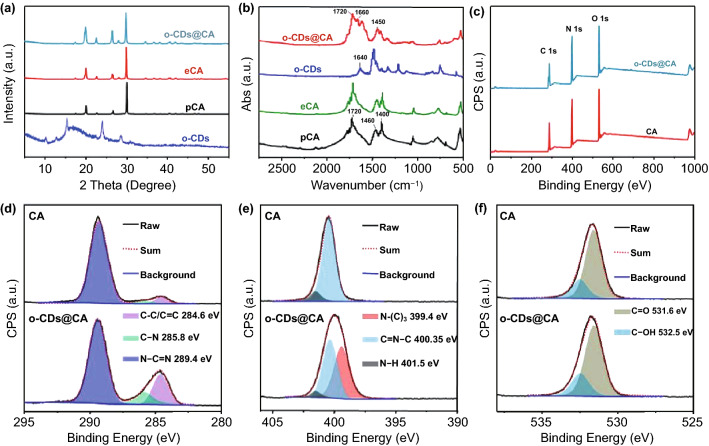
**a-b** XRD patterns and FT-IR spectra of o-CDs, pCA, eCA, and o-CDs@CA ( pCA: pure CA, purchased from the commercial source; eCA: experimental CA, prepared from urea by microwave method); **c** XPS spectra of CA and o-CDs@CA; **d-f** high resolution XPS spectra and the corresponding fitting curves of C 1s **d**, N 1s **e** and O 1s **f** of CA and o-CDs@CA

To verify the successfully embedding o-CDs into CA matrix, morphologies and phase structures of o-CDs and o-CDs@CA were characterized using TEM, SEM and XRD. As shown in Fig. S5, o-CDs are found to be monodispersed spherical nanoparticles with diameters of about 2–3 nm. The high resolution TEM (HR-TEM) image (insert of Fig. S5) and XRD analysis (Fig. 2a) indicate that o-CDs are mostly amorphous carbon or polymer-like structure with partially crystallized. Moreover, similar XRD patterns are observed from o-CDs@CA, pure CA (pCA, purchased from the commercial source) and experimental CA (eCA, prepared from urea by microwave method, see details in the section of Method) (Fig. 2a), indicating that fine CA crystals have been produced from urea via the microwave-assisted heating process either in the absence or presence of o-CDs. It is worthy to note that the intensities of some X-ray diffraction peaks of o-CDs@CA are found to be stronger than that of CA (Fig. 2a), probably attributing to the embedding of o-CDs into CA crystal would slightly affect the stacking of CA molecules and crystallization orientation. To further confirm successful embedding of o-CDs into CA, TEM and HR-TEM of o-CDs@CA were investigated. Although o-CDs are hardly observed from the TEM image (Fig. S6a), clear lattice fringes that corresponding to o-CDs can be found from their HR-TEM image (Fig. S6b), demonstrating that o-CDs have been successfully embedded into CA matrix. Note that the similarity of the XRD patterns and SEM images of o-CDs@CA and pCA (Figs. 2a andS7) indicate that the structure of CA can be mostly preserved even with o-CDs being embedded (probably due to the very low contents of o-CDs in CA).

In order to clarify the existence form of o-CDs in CA matrix, FT-IR and XPS measurements of o-CDs, CA and o-CDs@CA were carried out and systemically analyzed. It should be pointed out that CA is mainly stable as s-triazine-2,4,6-trione form in solid state while partly converts to s-triazine-2,4,6-triol form in aqueous solution (Figs. 1a and S8) [41, 42]. As a result, the characteristic peak observed at about 1720 cm^−1^ in the FT-IR spectra of o-CDs@CA, eCA and pCA should correspond to the stretching vibration of ketone carbonyl of CA (Fig. 2b). From comparing the FT-IR spectra of o-CDs@CA, eCA and free o-CDs, the increase in absorption at 1450 cm^−1^ and emerging absorption peak at about 1660 cm^−1^ are observed in o-CDs@CA spectrum, which are attributed to the stretching vibration of C-N bonds and C = O bonds of amides, respectively. These alterations imply that chemical reactions might have occurred during the microwave-assisted in situ embedding o-CDs into CA matrix with the formation of C-N covalent bonds between o-CDs and CA. Furthermore, the composition analysis based on XPS surveys indicated that the o-CDs@CA, o-CDs and CA are mainly consisted of the same elements (i.e., carbon, nitrogen, and oxygen) (Figs. 2c, S9 and Table S2). The increase in carbon contents in o-CDs@CA supports the successful embedding of o-CDs into CA. Moreover, the deconvoluted XPS spectra of C 1s, N 1s, and O 1s of o-CDs@CA and free CA were also performed and compared (Figs. 2d-f, S9 and Table S3). For o-CDs@CA, the C 1s spectrum can be deconvoluted into three binding energies at 284.6, 285.8, and 289.4 eV, corresponding to the C–C/C = C, C–N, and N–C = N bonds, respectively (Fig. 2d); the N1s XPS spectrum can be fitted with three binding energies at 399.4, 400.35, and 401.5 eV, which are attributed to the N-(C)_3_, C = N–C, and N–H bonds, respectively (Fig. 2e); and the O 1s XPS spectrum containing two components that can be assigned to C = O (531.6 eV) and C–OH (532.5 eV) bonds (Fig. 2f). The corresponding fitting results of these deconvoluted XPS spectra are summarized to provide relatively quantitative alterations of the chemical groups from CA to o-CDs@CA (Table S3). From which one can see obvious increases of C–C/C = C and C-N bonds components from CA to o-CDs@CA based on the C 1s fittings; significant decrease in N–H bonds and emergence of N-(C)_3_ bonds from CA to o-CDs@CA based on the N 1s fittings. These results clearly demonstrate the formation of C-N covalent bonds during in situ embedding o-CDs into CA matrix.

### Photophysical Properties of o-CDs@CA

**Fig. 3:**
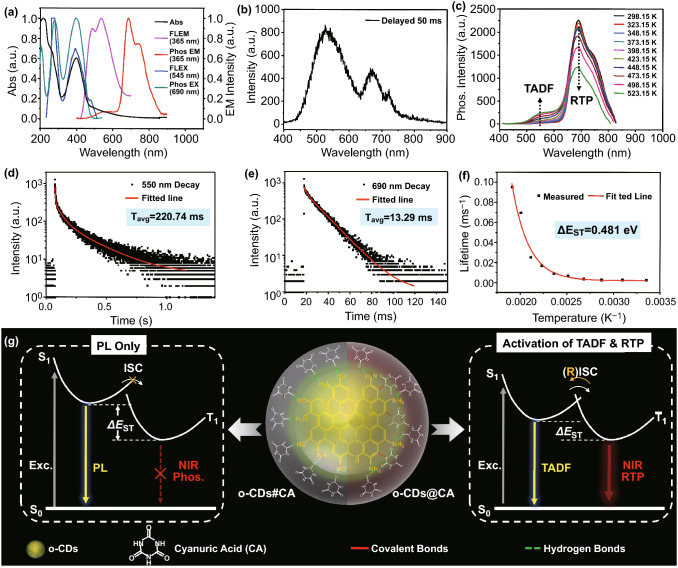
**a** Normalized UV–Vis absorption, photoluminescence (PL) emission (λ_EX_ = 365 nm) and excitation (λ_EM_ = 550 nm), and phosphorescence emission (λ_EX_ = 365 nm) and excitation (λ_EM_ = 690 nm) spectra of o-CDs@CA powder (Abs: absorption; EX/EM: excitation/emission); **b** afterglow emission spectrum of o-CDs@CA powder with 50 ms delay under the excitation of 365 nm at room temperature; **c** afterglow emission spectra of o-CDs@CA powder at different temperatures (TADF: thermal activated delayed fluorescence; RTP: room temperature phosphorescence); **d-e** afterglow decay spectra of o-CDs@CA powder monitored at 550 nm and 690 nm upon excitation of 365 nm; **f** afterglow lifetimes of o-CDs@CA as a function of temperature and fitting curve based on Eq. 2; **g** schematic illustration of the multiple fixation of o-CDs in CA matrix by covalent bonds, hydrogen bonds and physical confinements; possible emission processes of o-CDs embedded in CA by different strategies (Exc: excitation; ISC: intersystem crossing; RISC: reversed intersystem crossing)

Subsequently, photophysical properties of o-CDs@CA are fully investigated. As shown in Fig. 3a, the o-CDs@CA powder shows a strong absorption peak at 280 nm, a broad absorption band covering from ca. 300 to 500 nm, and PL emission at wavelength of maximum (λ_max_) 550 nm under the excitation of 365 nm. All these spectral data are similar to that of the dispersion of free o-CDs (Fig. S3), indicating that the PL emission of o-CDs@CA should be arisen from o-CDs. Significantly, the afterglow spectrum (Phos mode) of o-CDs@CA powder shows a dominating emission at wavelength of maximum 690 nm under the excitation of 365 nm (Fig. 3a, red line). To the best of our knowledge, this is the first report about the NIR afterglow of CDs-based materials. Note that this afterglow spectrum also contains a weak emission at shorter wavelength region from about 450 to 620 nm with the λ_max_ at ca. 550 nm. To demonstrate the inconsistence between afterglow color (green) and spectrum (dominating emission at λ_max_ = 690 nm) of o-CDs@CA powder, an afterglow spectrum with longer delay time (e.g., 50 ms) was measured, of which displaying stronger emission at 550 nm than 690 nm (Fig. 3b). Therefore, the longer afterglow lifetime at 550 nm and the insensitiveness of NIR emission to the human vision at 690 nm are believed to be responsible for the green afterglow of this material during directly observing (Fig. 1b, without filter). Certainly, the NIR afterglow at 690 nm (dark red) could be observed if an optical filter (λ_Cut-off_ = 600 nm) being used (Fig. 1b, with filter).

Based on the apparent similarities of the steady-state PL and short wavelength afterglow emission (from ca. 450 to 620 nm), and PL excitation spectrum at 550 nm and afterglow excitation spectrum at 690 nm (Fig. 3a), the two afterglow emission bands could be tentatively attributed to TADF (λ_max_ = 550 nm) and RTP (λ_max_ = 690 nm) of o-CDs@CA [35, 43]. To further confirm such an assumption, their temperature-dependent afterglow emission properties were investigated. As shown in Fig. 3c, it is observed a gradual enhancement of the afterglow intensity at 550 nm but decreases at 690 nm with the temperatures increasing from 298.15 to 523.15 K, demonstrating the nature of TADF and RTP at 550 and 690 nm, respectively [32, 34]. As shown in the afterglow decay spectra of o-CDs@CA, the RTP and TADF exhibit bi- and tri-exponential function decay processes, respectively, under the excitation of 365 nm (Fig. 3d-e and Table S4). According to Eq. 2 [38, 44]:

2 $$\tau_{{{\text{avg}}}} = {{\sum {\alpha_{i} \tau_{i}^{2} } } \mathord{\left/ {\vphantom {{\sum {\alpha_{i} \tau_{i}^{2} } } {\sum {\alpha_{i} } }}} \right. \kern-\nulldelimiterspace} {\sum {\alpha_{i} } }}\tau_{i}$$

the average lifetimes were calculated to be 220.74 ms for 550 nm of TADF and 13.29 ms for 690 nm of NIR RTP (under the excitation of 365 nm), which are in good argeement with the afterglow spectrum that measured by longer delay time (Fig. 3b). In general, a smaller *ΔE*_*ST*_ benefits the efficient intersystem crossing (ISC) process to promote Phos, but a too small *ΔE*_*ST*_ value (e.g., < 0.2 eV) would induce a very effective reversed ISC (RISC) process to populate a dominating TADF [44–47]. Therefore, tailoring energy gap *ΔE*_*ST*_ value to a suitable range is critical for simultaneously producing both Phos and TADF. The RISC determines the lifetime of TADF, so its decay rate constant (kRISC) can be used to estimate the *ΔE*_*ST*_ of a material according to Eq. 3:

3 $$k_{{{\text{RISC}}}} = A \cdot \exp \left( {{{ - \Delta E_{ST} } \mathord{\left/ {\vphantom {{ - \Delta E_{ST} } {k_{B} T}}} \right. \kern-\nulldelimiterspace} {k_{B} T}}} \right)$$

where *A* is a constant, *k*_*B*_ stands for the Boltzmann’s constant and *T* is the absolute temperature in Kelvin [48, 49]. On the basis of the temperature-dependent afterglow decay spectra of o-CDs@CA powder (Fig. S10 and Table S5), the decay rates can be plotted as a function of temperature and fitted based on the Eq. 3 (Fig. 3f and Table S6). Thus, the energy gap *ΔE*_*ST*_ of o-CDs@CA was calculated to be 0.481 eV, which is closely consistent with the estimated value of 0.478 eV from PL and Phos spectra measurements (Fig. S11). In contrast, the energy gap *ΔE*_*ST*_ of free o-CDs was determined to be 0.594 eV based on their low temperature PL and Phos spectra (Fig. S1), and it is evidently larger than that of the o-CDs@CA. Note that o-CDs only show very weak Phos emission at low temperature (77 K), which might attribute to their larger *ΔE*_*ST*_ and no effective ISC occurring. Consequently, the decrease in the energy gap *ΔE*_*ST*_ from o-CDs to o-CDs@CA should play a critical role to activate the dual-mode room temperature afterglow of o-CDs@CA (i.e., green TADF and NIR RTP).

### Dual-mode Afterglow Mechanism of o-CDs@CA

According to our previous study, formation of covalent bonds between CDs and matrices could induce a decrease in energy gap *ΔE*_*ST*_, being confirmed to be responsible for the dual-mode of afterglow emissions of the corresponding composites [32]. To experimentally confirm such an inference to be applicable to o-CDs@CA, another kind of composite of o-CDs and CA via co-crystallization (named o-CDs#CA, see details in the Experimental Section) was prepared and investigated. As shown in the Figs. S12-S14, the FT-IR and XPS spectra of this composite show no obvious differences in comparison with that of pCA, indicating that o-CDs are embedded and immobilized by CA matrix via non-covalent interactions (e.g., hydrogen bonds and physical confinements) [37]. Not surprisingly, no afterglow was observed from o-CDs#CA (Fig. S15), suggesting that hydrogen bonds between CA and o-CDs and physical confinements of CA to o-CDs are not sufficient to activate room temperature afterglow of o-CDs. In contrast, o-CDs@CA prepared from microwave-assisted in situ embedding o-CDs into CA matrix introduced covalent bonds between o-CDs and CA, offering an extra and more effective fixation and rigidification effects to the triplet states of o-CDs except for hydrogen bonds and physical confinements. Thus, the formation of covalent bonds between o-CDs and CA is believed to be critical for the dual-mode afterglows of o-CDs@CA. It is worth noting that although o-CDs@CA in solid state and dispersion state exhibit similar dual-mode afterglow emissions, both the TADF and NIR-RTP performances (lifetime and intensity) distinctly decreased in dispersion state (Fig. S4), indicating that hydrogen bonds and physical confinement effects should also provide some contributions to better fix and rigidify the triplet states of o-CDs in CA matrix. Finally, effects of molecular oxygen to the afterglow of o-CDs@CA are investigated. As shown in Fig. S16, the afterglow intensities were found to be nearly identical for o-CDs@CA powder under air and argon atmospheres, but about 25% higher under nitrogen than that of air-saturated condition for their aqueous dispersion. These findings demonstrate that the CA crystal could play a role to protect the excited states of o-CDs@CA from quenching by oxygen.

Based on the above discussion, mechanisms for activating the afterglow of o-CD@CA composite are proposed as follows. During microwave-assisted heating the mixture of urea and o-CDs, urea is gradually converting to CA and meanwhile embedding o-CDs through deamination and/or dehydration reactions (such processes resulting in the formation of C-N bonds and/or N-(C)_3_ structures). The formation of covalent bonds not only decreased the energy gap *ΔE*_*ST*_ from 0.594 eV of free o-CDs to 0.481 eV of o-CDs@CA, but also played a key role to stabilize the triplet states of o-CDs. Note that although the *ΔE*_*ST*_ of o-CDs decreased by formation covalent bonds with CA, it is still significantly larger than ideal values for efficiently producing TADF (i.e., < 0.2 eV) [45, 47]. Thus, the afterglow emission spectrum of o-CDs@CA exhibits a predominant NIR RTP and a relatively weak TADF (Fig. 3a, red line).

### Applications of the Dual-mode Afterglow from o-CDs@CA

**Fig. 4:**
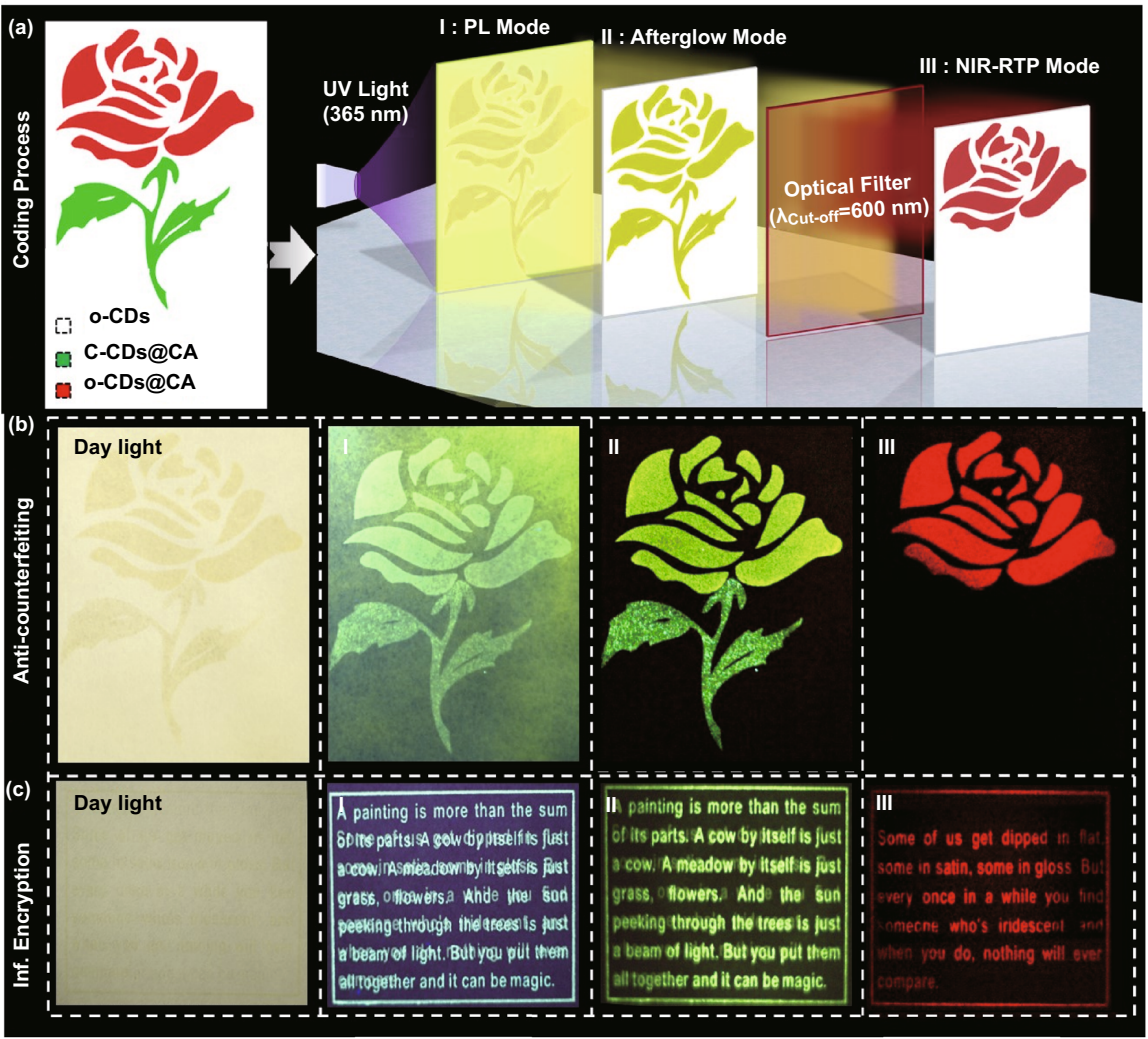
**a** Schematic illustration of the encryption and decryption procedure using the o-CDs@CA in information security applications. The o-CDs are directly dying on the filter paper, the o-CDs@CA and C-CDs@CA are dispersed in commercial ink and fabricated onto the filter paper by silk-screen printing); **b-c** the images of the interference patterns (I–II) (under PL and afterglow modes) and decrypted pattern (III) (afterglow mode with the using of an optical filter (λ_Cut-off_ = 600 nm)) for anti-counterfeiting **b** and information (Inf.) encryption **c**

Interestingly, although the green TADF emission of o-CDs@CA is relatively weaker than their NIR RTP, the NIR RTP is found to completely covered by the green TADF during directly observing (Fig. 1b, without filter). This phenomenon could be attributed to the shorter lifetime of the NIR RTP and the insensitive/invisible feature of NIR light to human vision. As a result, the o-CDs@CA powder only displays a green afterglow either observed by the naked eye or acquired images by a camera. However, the NIR RTP emission can be readily observed if an optical filter (λ_Cut-off_ = 600 nm) being used (Fig. 1b, with filter). Inspired by these unique afterglow properties and high stability (Figs. S17 and S18), o-CDs@CA are believed to be very attractive in information security fields. To demonstrate such potentials, anti-counterfeiting and information encryption applications of this material were preliminary investigated. As shown in Fig. 4a, background of a substrate is firstly pre-dyed using o-CDs, and then the security pattern, a rose for example, is printed using two components: o-CDs@CA for the flower and the C-CDs@CA (C-CDs being prepared from citric acid and embedded into CA matrix using the same procedure as that of o-CDs@CA, see experimental section in Method for details) for the leaves and stems. Note that this pattern can be fabricated/printed onto filter paper and many other substrates through a simple silk-screen printing technique by prior dispersing the composites in commercial ink. Under the PL mode, the whole background displays a green emission under the irradiation of 365 nm UV lamp (Fig. 4b-I), making the printed pattern difficult to be recognized by the naked eye. To the afterglow mode, a long-lived green emission is observed from the whole printed rose that lasting for several seconds after ceasing the UV lamp ((Fig. 4b-II, and S19). Importantly, only the flower part that printed using o-CDs@CA is observed with an optical filter (λ_Cut-off_ = 600 nm) being used under the afterglow mode (dark red, arising from the NIR RTP of o-CDs@CA, Fig. 4b-III). Moreover, high-level information encryption could also be realized using the similar strategy, i.e., the concealed information being able to recognize only under the afterglow mode with using an optical filter (λ_Cut-off_ = 600 nm) (Fig. 4c). Therefore, the o-CDs@CA composite coded information could be well concealed by visible light emission whether under PL mode or afterglow mode, making the security patterns very difficult to be forged and mimicked. These results clearly demonstrate a great potential of o-CDs@CA in advanced security applications through a special manner for information readout.

### Universality of the Method for Preparing CDs@CA Afterglow Materials

**Fig. 5:**
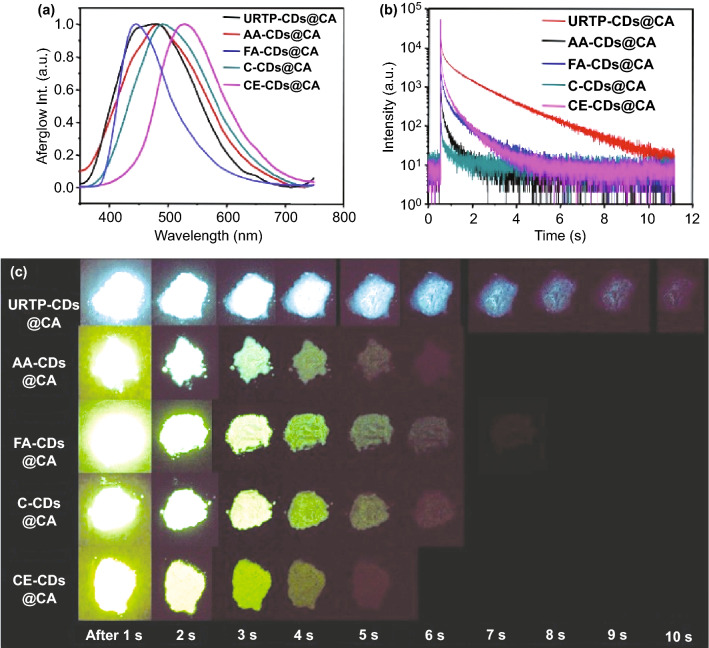
**a** Normalized afterglow emission spectra of various kinds of CDs@CA composites; **b** the afterglow decay spectra of various kinds of CDs@CA composites; **c** photographs of various kinds of CDs@CA composites in solid state after ceasing the 365 nm UV irradiation (1–10 s) under ambient conditions

Finally, universality of the as-developed in situ embedding and fixing method for activating room temperature afterglow of CDs was examined. To perform this, a variety of CDs (e.g., URTP-CDs, AA-CDs, FA-CDs, C-CDs and CE-CDs, see the details of their synthesis in the section of Method) are selected and treated as that of for o-CDs@CA. As expected, all these CDs-based composites exhibit distinct afterglow at ambient conditions under 365 nm UV light irradiation, no matter in solid state (Fig. 5) or dispersion state (Fig. S20). Notably, although the free URTP-CDs and AA-CDs show RTP property [24, 29], their afterglow lifetimes become obviously longer after embedding in CA matrix. In addition, the afterglow performances of all these CDs-based composites are found to be improved in comparison with the corresponding composites prepared by other methods, e.g., prolonging afterglow lifetimes and/or extending the occurrence of afterglow from only solid state to aqueous dispersion state (Figs. S20 and S21 and Table S7). The universality of this method might be ascribed to containing abundant –OH and/or –NH_2_ functional groups on these CDs (Fig. S22), offering possible reaction sites to form covalent bonds with CA during its production process. The formation of covalent bonds between CDs and CA is considered to be critical for observation their afterglow even in aqueous dispersions [32, 50]. Overall, the strategy developed in this study is applicable to many other kinds of CDs, which provides not only a better choice to fix CDs for activating their room temperature afterglow, but also a method to achieve afterglow in aqueous media.

## Conclusions

In summary, a facile method is developed to achieve green TADF and NIR RTP dual-mode afterglow emissions from the composite of o-CDs and CA (i.e., o-CDs@CA). Through in-depth discussion, we reveal that the formation of covalent bonds between o-CDs and CA plays critical roles for activating the dual-mode afterglows, which not only appropriately decreased the energy gap *△E*_*ST*_ of o-CDs to facilitate both ISC and RISC processes, but also effectively fixed and rigidified the triplet states of o-CDs. Besides, hydrogen bonds between o-CDs and CA and physical confinements of the CA matrix to o-CDs would also contribute some effects to better stabilize the triplet species of o-CDs. Due to the shorter lifetime of NIR RTP of o-CDs@CA and the invisibility/insensitivity of NIR light to human vision, the NIR RTP is completely covered by the green TADF during directly observing. The hidden NIR emission, however, can be readily captured if an optical filter (λ_Cut-off_ = 600 nm) being applied. By utilizing these unique features, o-CDs@CA are demonstrated to be excellent confidentiality in anti-counterfeiting and information encryption applications. Finally, universality of the as-developed method is confirmed to be applicable to many other kinds of CDs for achieving or enhancing their afterglow performance. Since the as-developed method could extend the occurrence of afterglow of CDs to aqueous media, this would be significant in biologically relevant applications, and such work is now undergoing in our lab.

## Supplementary Information

Below is the link to the electronic supplementary material.

Supplementary file1 (PDF 1538 kb)
