# Supplementary material for: Green and Near-Infrared Dual-Mode Afterglow of Carbon Dots and Their Applications for Confidential Information Readout
Source: Nanomicro Lett. 2021 Sep 16;13:198. doi: 10.1007/s40820-021-00718-z (PMC8446126; doi:10.1007/s40820-021-00718-z)
Supplement: Supplementary file 1 — Supplementary file1 (PDF 1538 kb) [file 40820_2021_718_MOESM1_ESM.pdf]

Supporting Information for

Green and Near-Infrared Dual-Mode Afterglow of Carbon Dots and Their Applications for Confidential Information Readout

Yuci Wang[1], Kai Jiang[2, 3, *], Jiaren Du[2], Licheng Zheng[2], Yike Li [1, *], Zhongjun Li[1], Hengwei Lin[2, *]

[1]College of Chemistry, Zhengzhou University, Zhengzhou 450001, P. R. China

[2] International Joint Research Center for Photo-responsive Molecules and Materials, School of Chemical and Material Engineering, Jiangnan University, Wuxi 214122, P. R. China

[3] Key Laboratory of Graphene Technologies and Applications of Zhejiang Province, Ningbo Institute of Materials Technology & Engineering (NIMTE), Chinese Academy of Sciences, Ningbo 315201, P. R. China

*Corresponding authors. E-mail: jiangkai@nimte.ac.cn (Kai Jiang); liyike@zzu.edu.cn (Yike Li); linghengwei@jiangnan.edu.cn (Hengwei Lin)

Supplementary Figures and Tables

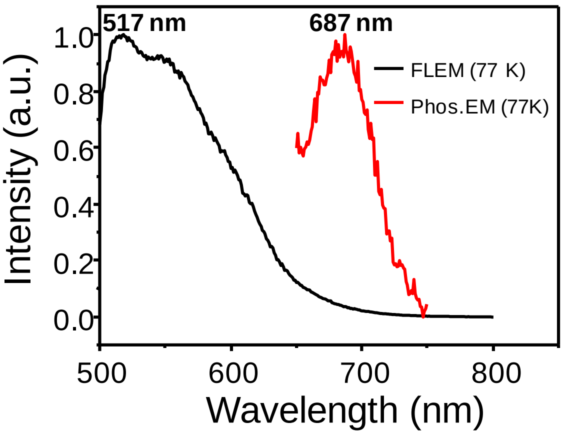

Fig. S1 Low temperature (77 K) fluorescence (black line) and phosphorescence (red line) emission spectra of o-CDs dispersed in ethanol

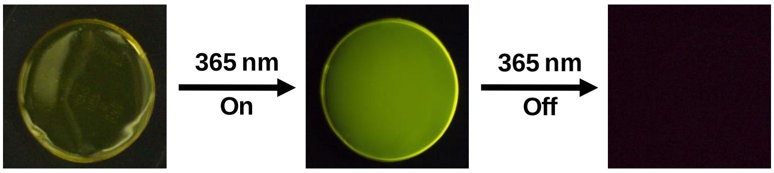

Fig. S2 Photographs of o-CDs-PVA film under daylight, with the irradiation of 365 nm UV lamp and after the lamp was switched off under ambient conditions

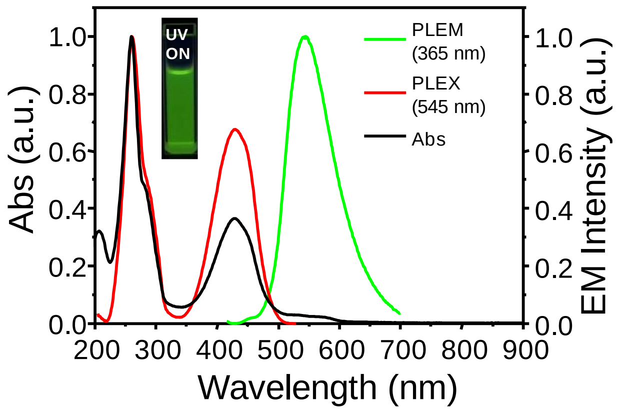

Fig. S3 UV-Vis absorption, FL emission and excitation spectra of the o-CDs dispersion in ethanol (inset: FL emission image of the o-CDs ethanol dispersion under 365 nm excitation)

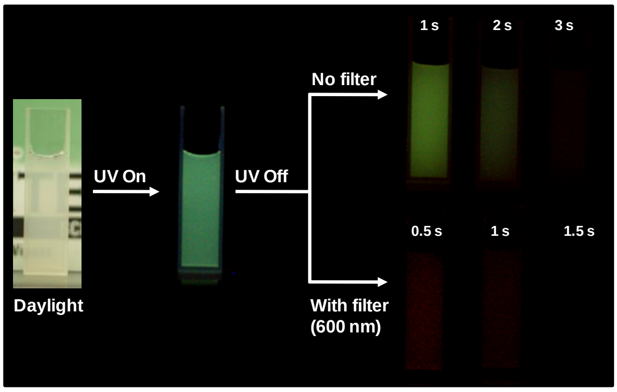

Fig. S4 Photographs of the afterglow emission of o-CDs@CA water dispersion under daylight and with the 365 nm UV lamp of irradiation on and off

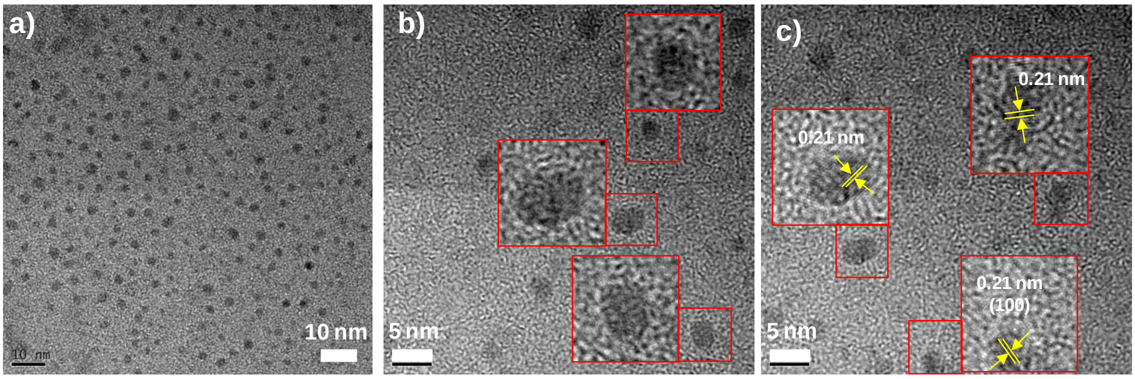

Fig. S5 a) TEM, and **b-c**) high resolution TEM images of o-CDs (the larger red squares in b and c being enlarged images of the nearby smaller squares)

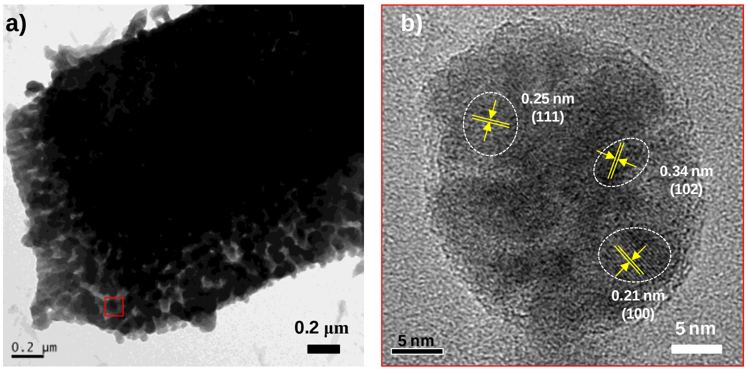

Fig. S6 a) TEM, **b**) high resolution TEM (red area) images of the o-CDs@CA

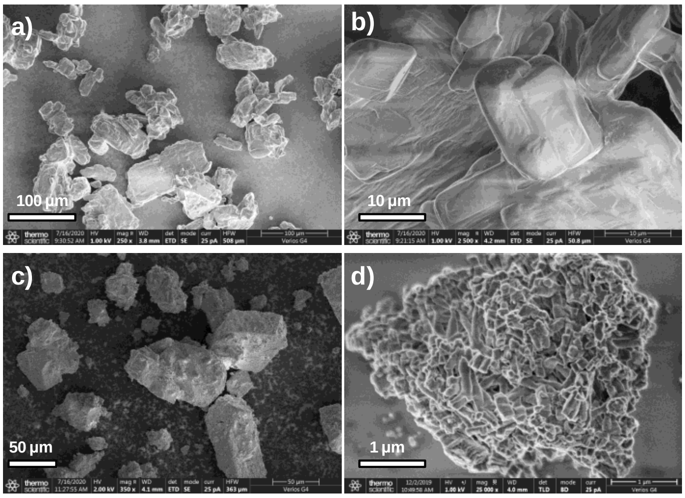

Fig. S7 SEM images of pCA (**a-b**) and o-CDs@CA (**c-d**)

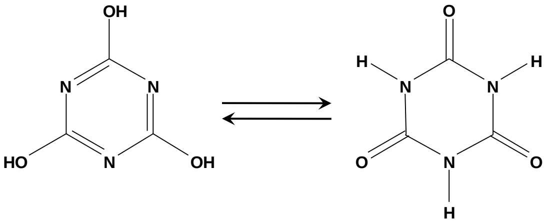

s-triazine-2,4,6-triol s-triazine-2,4,6-trione

Fig. S8 Schematic illustration of the structural interconversion of CA from solution state (left) to solid state (right)

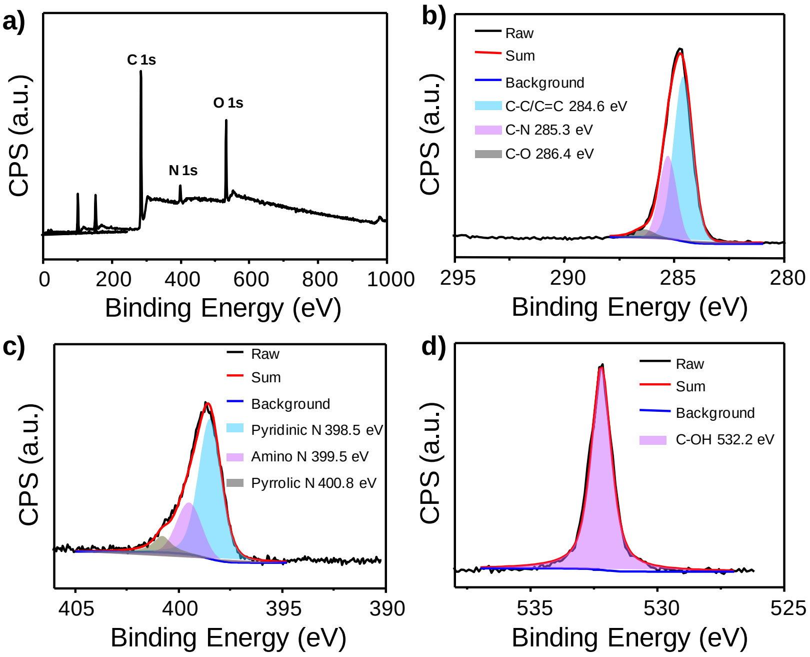

Fig. S9 XPS survey (**a**), high resolution C 1s (**b**), N 1s (**c**) and O 1s (**d**) spectra of o-CDs

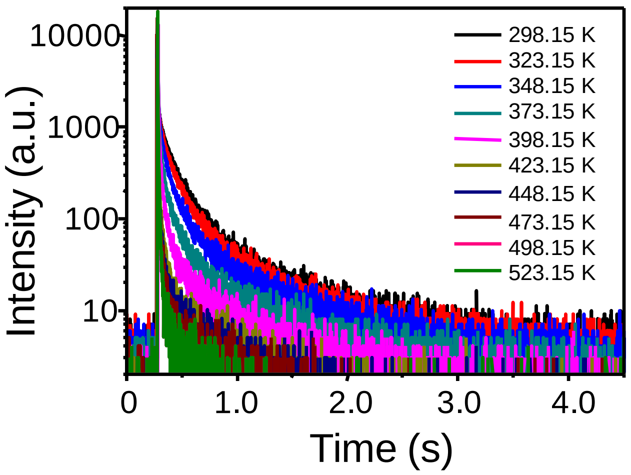

Fig. S10 Afterglow decay profiles (λ_{em}=550 nm) of o-CDs@CA powder at 298.15 to 523.15 K under 320 nm excitation

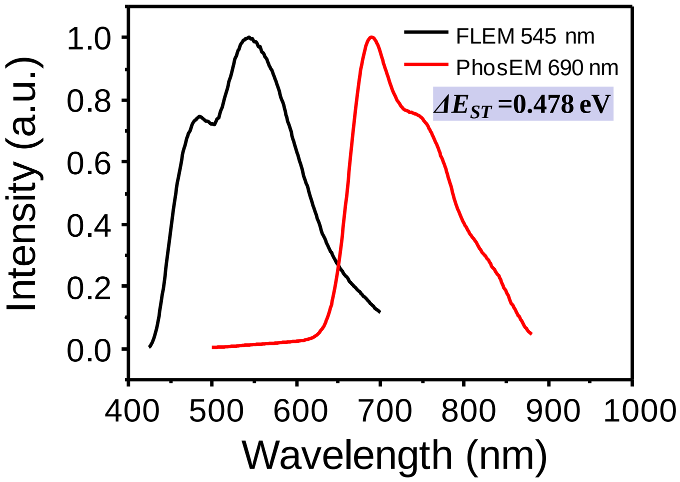

Fig. S11 Fluorescence and phosphorescence emission spectra (λ_{exc}=400 nm) of o-CDs@CA powder for the calculation of its energy gap $\triangle E_{ST}$

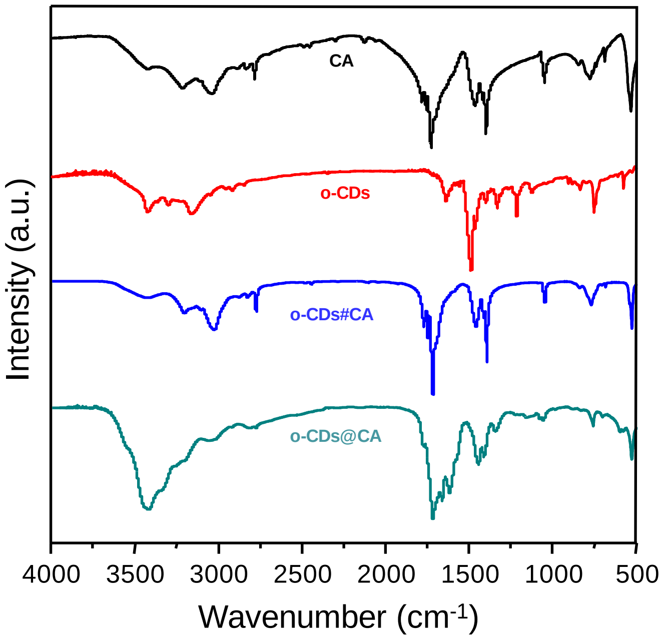

Fig. S12 FT-IR spectra of CA, o-CDs, o-CDs@CA and o-CDs#CA

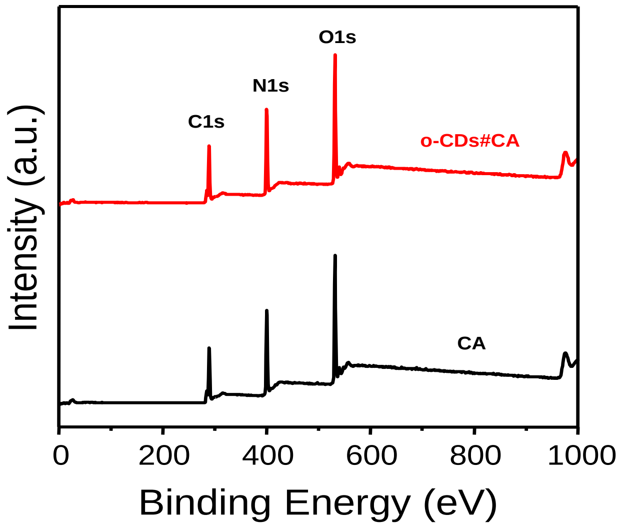

Fig. S13 XPS surveys of CA and o-CDs#CA

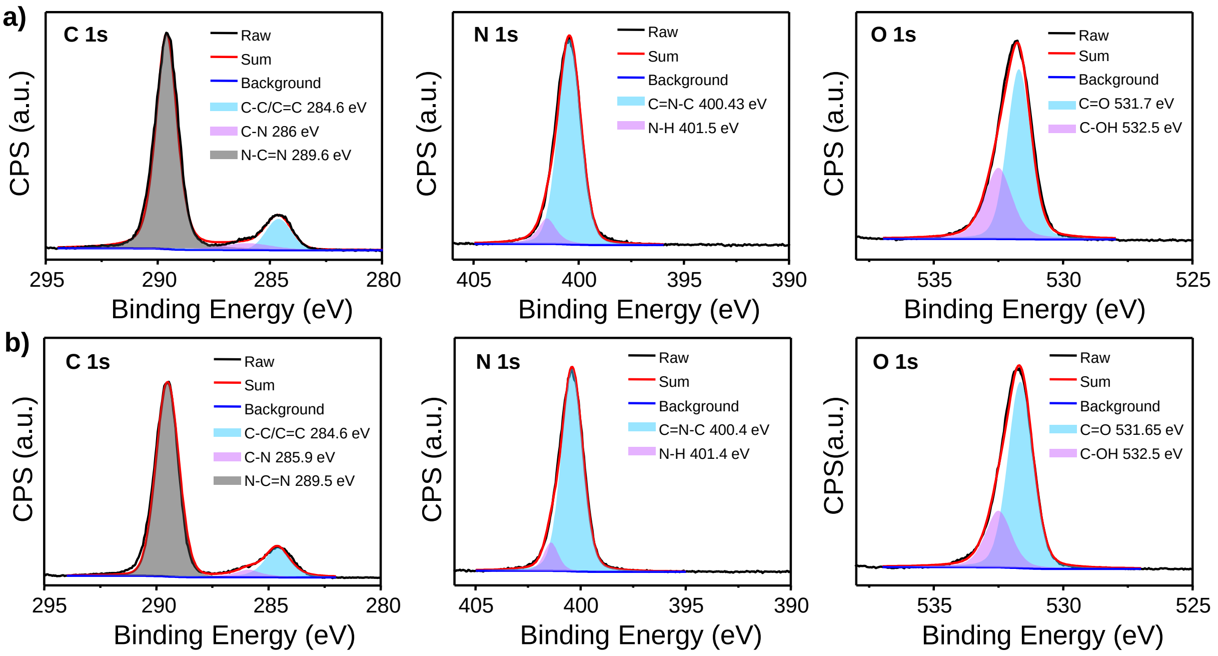

Fig. S14 High resolution XPS spectra of C1s, N1s and O1s of CA (**a**) and o-CDs#CA (**b**)

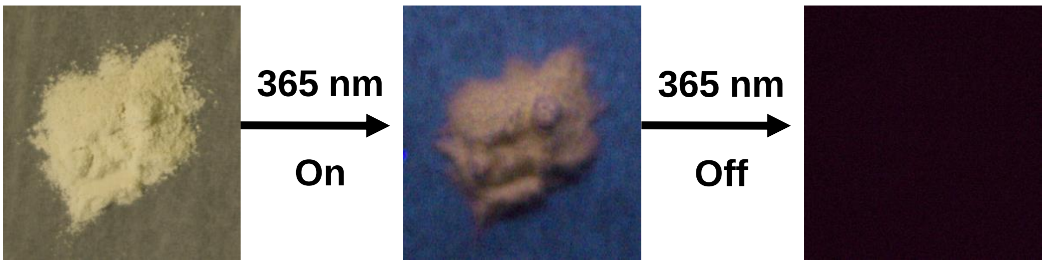

Fig. S15 Photographs of o-CDs#CA powder under daylight, and irradiation with 365 nm UV lamp ON and after the lamp was just switched off

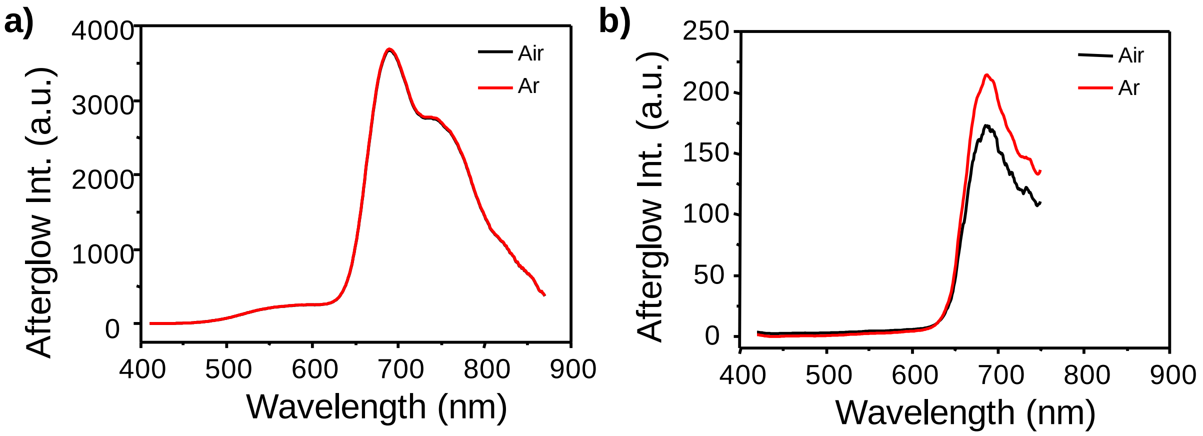

Fig. S16 Afterglow emission spectra of **a**) o-CDs@CA powder under air (Air) and argon (Ar) atmospheres at 365 nm excitation, **b**) o-CDs@CA under air-saturated (Air) and argon (Ar) conditions in water dispersion at 400 nm excitation

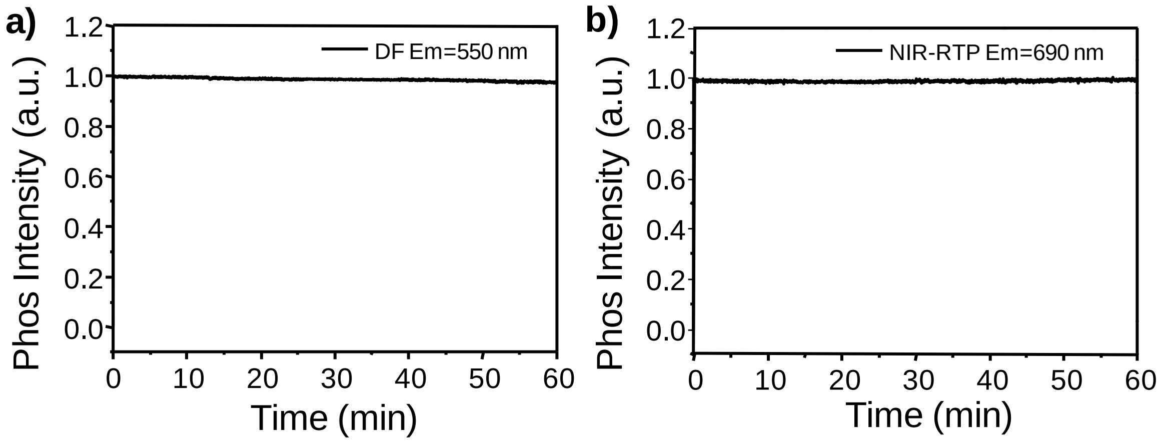

Fig. S17 Photostability of the o-CDs@CA powder under continuous excitation at 365 nm (emission wavelength of 550 nm and 690 nm) for one hour using spectrofluorometer equipped with a xenon lamp (150 W)

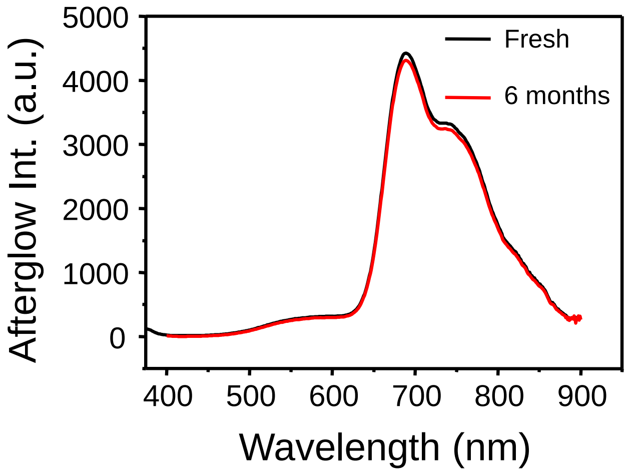

Fig. S18 Comparison of the afterglow emission of the freshly-prepared and the stored (6 months) o-CDs@CA powder under excitation of 365 nm

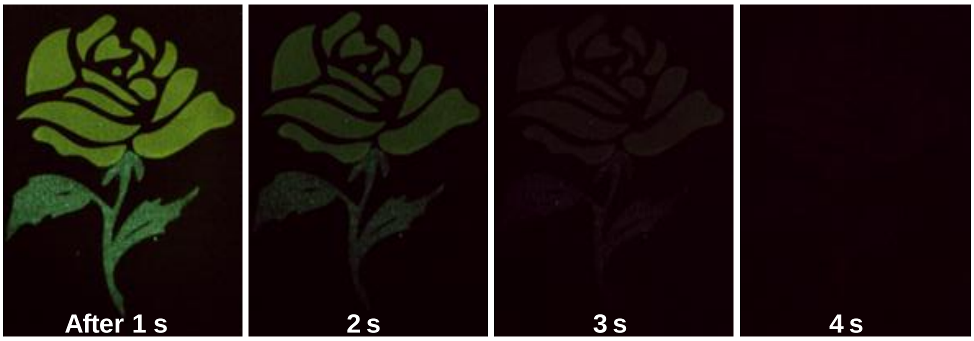

Fig. S19 Photographs of o-CDs@CA ink patterned flower after switching off the 365 nm UV lamp

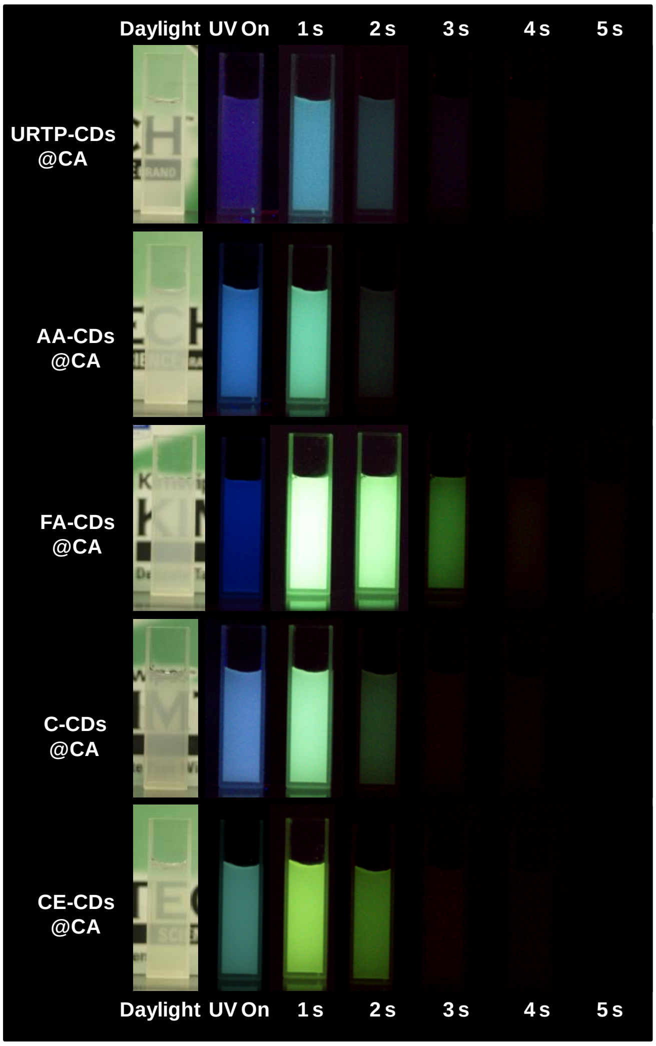

Fig. S20 Photographs of the URTP-CDs@CA, AA-CDs@CA, FA-CDs@CA, C-CDs@CA and CE-CDs@CA water dispersions under daylight, and with irradiation of 365 nm UV lamp on and off

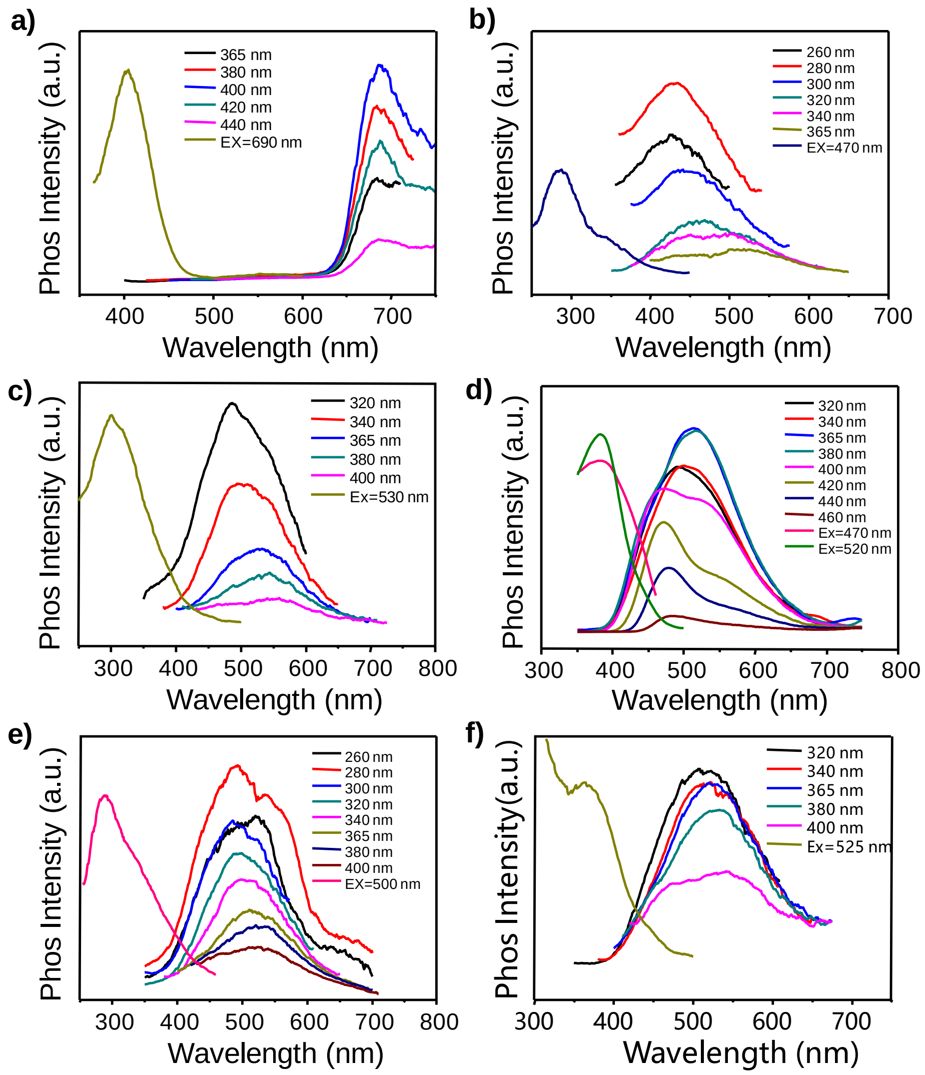

Fig. S21 Afterglow emission and excitation spectra of **a**) o-CDs@CA, **b**) URTP-CDs@CA, **c**) AA-CDs@CA, **d**) FA-CDs@CA, **e**) C-CDs@CA, and **f**) CE-CDs@CA in water dispersion

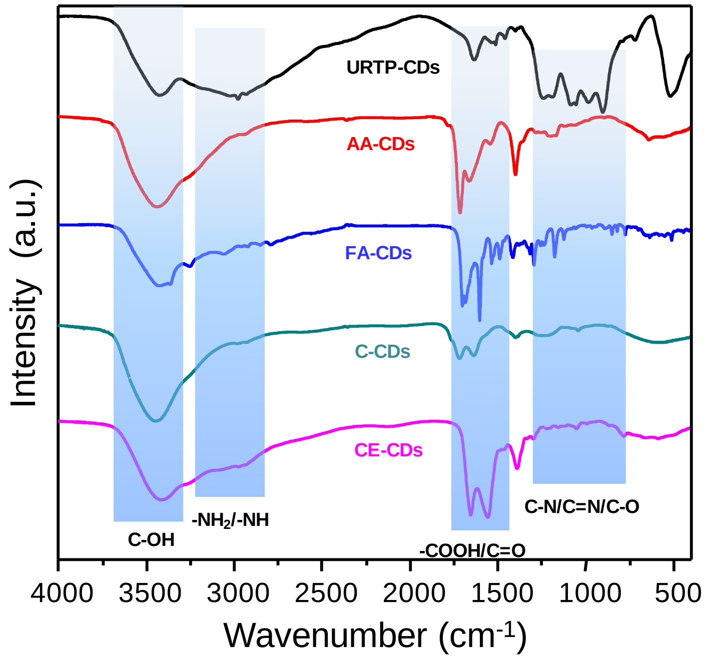

Fig. S22 FT-IR spectra of URTP-CDs, AA-CDs, FA-CDs, C-CDs and CE-CDs

Table S1 PL quantum yields (QYs) of o-CDs@CA composites prepared by different ratios of o-CDs and urea

**ratio of o-CDs to urea (w/w, %)**	0.005 %	0.0125 %	0.05 %	0.1 %	0.25 %	0.5 %
**QYs**	1.95	2.42	3.00	2.87	2.07	1.35

NOTE: Although the PL QY of 0.1% ratio is slightly lower than 0.05% ratio, it was observed that the 0.1% ratio sample exhibited better PL and afterglow performances. Therefore, 0.1% was selected as the optimal ratio to prepare o-CDs@CA and discussed in this study.

Table S2 Relative contents of C, N and O atoms for o-CDs, CA, o-CDs@CA and o-CDs#CA (determined by XPS survey)

**Sample**	**C (%)**	**N (%)**	**O (%)**
**o-CDs**	78.99	12.87	8.14
**CA**	37.58	38.17	24.25
**o-CDs@CA**	41.58	36.11	22.31
**o-CDs#CA**	40.90	31.40	27.69

Table S3 Relative contents of different functional groups in CA, o-CDs@CA and o-CDs#CA (based on the fitting results of Fig. 2d-f and Fig. S14)

**Sample**	**C 1s (%)**			**N 1s (%)**			**O 1s (%)**	
	**C-C/C=C**	**C-N**	**N-C=N**	**N-(C)$_3$**	**C=N-C**	**N-H**	**C=O**	**-OH**
**CA**	12.09	6.59	81.32	-	88.85	11.15	65.40	34.60
**o-CDs@CA**	21.26	12.70	66.04	47.91	46.92	5.17	74.93	25.07
**o-CDs#CA**	15.89	4.87	79.24	-	90.69	9.31	74.79	25.21

Table S4 Fitting results of the afterglow lifetimes of the o-CDs@CA powder at 550 and 690 nm under 320 nm excitation

**λ_{ex} [nm]**	**λ_{em} [nm]**	**B_1 [%]**	**τ_1 [ms]**	**B_2 [%]**	**τ_2 [ms]**	**B_3 [%]**	**τ_3 [ms]**	**τ_{avg} [ms]**	**χ^2**
365	550	6.46	4.69	27.63	49.90	65.91	236.29	**220.74**	1.1655
365	690	5.55	2.26	94.45	13.4	-	-	**13.29**	1.1296

Table S5 Afterglow lifetimes of the o-CDs@CA powder at different temperatures (λ_{ex}=320 nm, λ_{em}=550 nm, based on the afterglow decay curves in Fig. S10)

T (K)	λ_{ex} [nm]	λ_{em} [nm]	B_1 [%]	τ_1 [ms]	B_2 [%]	τ_2 [ms]	B_3 [%]	τ_3 [ms]	τ_{avg} [ms]	χ^2
**298.15**	320	550	55.37	108.88	34.98	522.04	9.65	9.744	**417.91**	1.327
**323.15**	320	550	52.14	91.98	36.68	472.9	11.18	7.9167	**388.87**	1.302
**348.15**	320	550	47.81	65.80	36.82	441.8	15.37	5.1188	**379.35**	1.550
**373.15**	320	550	44.13	45.38	19.34	3.556	36.53	349.9	**307.24**	1.439
**398.15**	320	550	46.41	16.66	35.69	165.1	17.89	1.668	**147.23**	1.635
**423.15**	320	550	54.07	10.69	28.86	129.8	17.07	0.9188	**113.47**	1.278
**448.15**	320	550	60.83	6.978	23.41	73.49	15.76	0.704	**60.01**	1.397
**473.15**	320	550	66.41	5.589	18.08	53.74	15.51	0.5868	**40.16**	1.323
**498.15**	320	550	17.76	0.5763	68.03	4.320	14.21	23.61	**14.38**	1.656
**523.15**	320	550	17.88	0.5295	69.45	3.894	12.67	18.52	**10.50**	1.285

Table S6 Afterglow lifetimes of various CDs before and after compositing with different matrices

Sample	Matrix	λ_{ex} [nm]	λ_{em} [nm]	B_1 [%]	τ_1[s]	B_2 [%]	τ_2[s]	B_3 [%]	τ_3[s]	τ_{avg}[s]	χ^2	Refs.
**URTP-CDs**	-	-	-	-	-	-	-	-	-	**1.46**		[S1]
	CA	355	520	19.83	0.315	74.88	1.640	5.18	0.0292	**1.57**	1.793	
	PVA	355	530	32.19	0.405	62.63	1.306	5.18	0.0430	**1.18**	1.537	
**AA-CDs**	-	-	-	-	-	-	-	-	-	**0.24**		[S2]
	CA	300	485	35.23	0.0791	49.56	0.526	15.22	0.00573	**0.48**	1.310	
	PVA	300	485	47.19	0.102	28.89	0.523	23.92	0.0164	**0.41**	1.682	
**FA-CDs**	CA	300	445	37.29	0.155	57.90	1.025	4.81	0.0108	**0.95**	1.305	
	PVA	300	540	30.23	0.249	58.73	0.976	11.04	0.0354	**0.89**	1.002	
	Biuret	-	-	-	-	-	-	-	-	**0.93**		[S3]
**C-CDs**	CA	300	490	22.98	0.035	66.96	0.452	11.07	0.00385	**0.44**	1.107	
	PVA	365	545	41.99	0.154	45.23	0.638	12.78	0.0201	**0.55**	1.433	
	BA	-	-	-	-	-	-	-	-	**1.6**		[S4]
**CE-CDs**	CA	320	530	40.45	0.137	44.13	0.713	15.42	0.0181	**0.62**	1.505	
	PVA	300	535	52.55	0.163	16.92	0.027	30.52	0.620	**0.47**	1.596	

Supplementary References

[S1] K. Jiang, Y. Wang, X. Gao, C. Cai, H. Lin, Facile, quick, and gram-scale synthesis of ultralong-lifetime room-temperature-phosphorescent carbon dots by microwave irradiation. Angew. Chem. Int. Ed. **57**(21), 6216-6220 (2018). https://doi.org/10.1002/anie.201802441

[S2] S. Hu, K. Jiang, Y. Wang, S. Wang, Z. Li et al., Visible-light-excited room temperature phosphorescent carbon dots. Nanomaterials **10**(3), 464 (2020). https://doi.org/10.3390/nano10030464

[S3] Q. Li, M. Zhou, Q. Yang, Q. Wu, J. Shi et al., Efficient room-temperature phosphorescence from nitrogen-doped carbon dots in composite matrices. Chem. Mater. **28**(22), 8221-8227 (2016). https://doi.org/10.1021/acs.chemmater.6b03049

[S4] W. Li, W. Zhou, Z. Zhou, H. Zhang, X. Zhang et al., A universal strategy for activating the multicolor room-temperature afterglow of carbon dots in a boric acid matrix. Angew. Chem. Int. Ed. **58**(22), 7278-7283 (2019). https://doi.org/10.1002/anie.201814629
